# Effects of *Lactobacillus kefiranofaciens* M1 Isolated from Kefir Grains on Germ-Free Mice

**DOI:** 10.1371/journal.pone.0078789

**Published:** 2013-11-11

**Authors:** Yen-Po Chen, Ming-Ju Chen

**Affiliations:** 1 Department of Animal Science and Technology, National Taiwan University, Taipei, Taiwan, ROC; 2 Center for Biotechnology, National Taiwan University, Taipei, Taiwan, ROC; University of California Merced, United States of America

## Abstract

*Lactobacillus kefiranofaciens* M1 is a novel probiotic strain that was isolated from kefir grains. Previously, we have demonstrated the immunoregulatory, anti-allergic, anti-asthmatic and anti-colitis abilities of *L. kefiranofaciens* M1 in a number of *in-vitro* and *in-vivo* experiments. However, whether the effects of *L. kefiranofaciens* M1 are elicited directly on the host or act by regulating the host's microbiota remains unknown. A number of studies have used germ-free or gnotobiotic animals to investigate the relationship between probiotics and colitis; therefore the aim of this study was to investigate the effects of *L. kefiranofaciens* M1 on germ-free mice. Such an approach should help in determining the direct effects of *L. kefiranofaciens* M1 on the host itself. Four-week-old female germ-free mice were inoculated intragastrically with 2×10^8^ CFU/mouse *L. kefiranofaciens* M1 once or at 2-day intervals for 14 days. Bacterial colonization, the Th1/Th2 cytokine profile of the mice's splenocytes and the anti-colitis effect of *L. kefiranofaciens* M1 were investigated. The strongest response in terms of splenic Th1 cytokine IFN-γ and IL-12 production upon TLR activation was detected in the continuous treatment group when comparing to the single inoculation group and the germ-free control. In addition, continuous inoculation with *L. kefiranofaciens* M1 was found to ameliorate the symptoms of DSS-induced colitis in germ-free mice. However, *L. kefiranofaciens* M1 failed to colonize the host. Thus it would seem that *L. kefiranofaciens* M1 is likely to act directly on the host and not be involved in microbiota regulation.

## Introduction

Kefir is a traditional alcoholic dairy beverage fermented using kefir grains; it is composed of various species of bacteria and yeasts that have symbiotic relationships with each other [1]. Recently, studies targeting kefir have come to the attention of the public because of kefir's potential health promoting effects, which include antimicrobial activity, anticarcinogenic properties, antimutagenic properties, improved gastrointestinal functioning, cholesterol lowering effects and immunoregulatary effects [2]. However, it is hard to standardize the composition and probiotic effects of kefir because the microbiota population of kefir grains is dynamic in nature and can be affected by the source of the milk, the production process and various other factors that may influence the composition of the kefir grains [3]. Accordingly, it was important and necessary to isolate and investigate in depth the potential probiotic bacteria/yeasts present in kefir grains that are responsible for its health promoting effects.

*Lactobacillus kefiranofaciens*, a major bacterial species found in kefir and kefir grains [2], [4]–[6], is one of the active constituent responsible for the health benefits of milk kefir [2]. Recently, we isolated a novel probiotic strain, *Lactobacillus kefiranofaciens* M1, from Taiwanese kefir grains [4]. This strain has been shown to have *in-vivo* anti-allergic and anti-asthmatic effects using an ovalbumin-induced allergic mouse models [7], [8]. *L. kefiranofaciens* M1 also has been demonstrated to enhance intestinal epithelial barrier function *in vitro* and protect against both dextran sodium sulfate (DSS) [9] and enterohemorrhagic *Escherichia coli* induced colitis in mice (unpublished data). In addition, the possible mechanisms involved in the health benefits provided by *L. kefiranofaciens* M1 have been investigated. This strain has been demonstrated to have an immunity enhancing ability that occurs via activation of macrophages beneath the Peyer's patch, which, in turn, induced the production of various T helper (Th) 1 cytokines (IL-1β, IL-6, IL-12 and TNF-α) and CD4^+^CD25^+^ Tregs via the Toll-like receptor (TLR) 2 [10]. However, whether the probiotic effects of *L. kefiranofaciens* M1 occur via a direct effect on the host or via the regulation of the host's microbiota in intestine remains unknown.

In order to study intestinal host-microbe interactions without interference from the complex intestinal microbiota and microbes that are not of direct interest [11], germ-free animals have been used in many studies of human disease and microbiota including intestinal host-microbe interactions [12]. Nonetheless, few studies using germ-free or gnotobiotic animals have focused on the relationship between use of a probiotic and colitis. Thus, in the current study, we have used germ-free mice mono-associated with *L. kefiranofaciens* M1 to elucidate the role of *L. kefiranofaciens* M1 in its immunoregulatory and anti-colitic effects on mice without interference from other host-microbe interactions.

## Materials and Methods

### *Lactobacillus kefiranofaciens* M1 preparation

*Lactobacillus kefiranofaciens* M1 (2×10^6^ CFU) was cultured in 10 mL deMan, Rogosa and Sharp (MRS) broth (Difco Laboratories, Detroit, MI) at 30°C for 36 hours (log phase; O.D. value  = 0.8) and harvested by washing and resuspending three times in phosphate buffered saline (PBS) (Hyclone, South Logan, UT). After washing, the cells were resuspended in PBS and adjusted to the desired concentration.

### Gnotobiotic mice generation and *L. kefiranofaciens* M1 treatment

**Figure 1:**
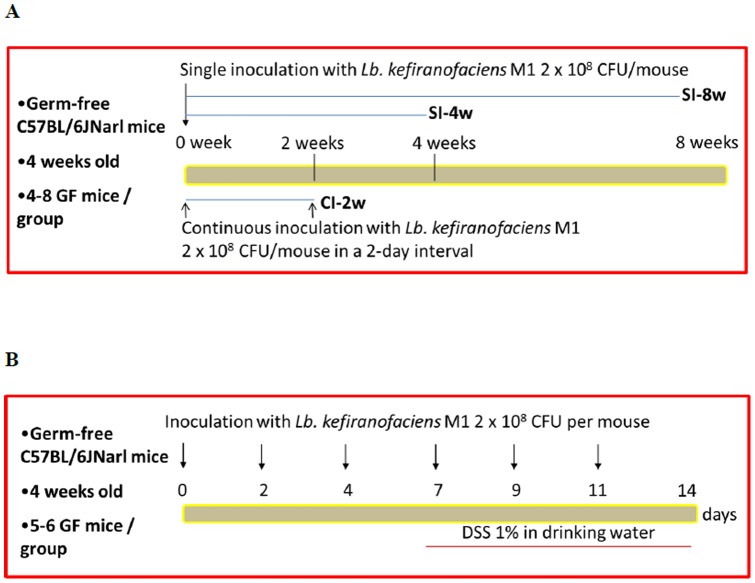
Inoculation scheme of *Lactobacillus kefiranofaciens* M1 for (A) gnotobiotic mice generation and (B) dextran sodium sulfate (DSS)-induced colitis.

Germ-free mice were maintained in sterilized vinyl isolators and kept at a constant temperature (20–26°C) and humidity (40–60%) in National Laboratory Animal Center (Taipei, Taiwan). Four-week-old female germ-free C57BL/6JNarl mice were administrated intragastrically with control sterilized PBS or 2×10^8^ CFU/mouse live *L. kefiranofaciens* M1 once. Four or eight weeks after this single inoculation (the SI-4w and SI-8w groups, respectively, Fig. 1A), the treated mice were sacrificed and target organs were collected for analysis. In parallel, 4-week-old female germ-free C57BL/6JNarl mice were inoculated intragastrically with control sterilized PBS or 2×10^8^ CFU/mouse live *L. kefiranofaciens* M1 repeatedly at 2-day intervals for 2 weeks (the CI-2w group, Fig. 1B). These mice were sacrificed at the end of intragastric inoculation with *L. kefiranofaciens* M1 and target organs were collected for analysis.

### Ethics Statement

All animal experiments were approved by Institutional Animal Care and Use Committee in National Laboratory Animal Center and performed in accordance with guidelines for animal care of the National Science Council in Taiwan (IACUC approval number IACUC2011H01).

### Fecal lactic acid bacteria count

The mouse feces were collected at the indicated intervals under aseptic condition for microbiological analysis. The collected feces were weighted, resuspended by vortex and then serial diluted in 0.85% sodium chloride (Sigma, St Louis, MO, USA) solution. The colony forming units (CFU) of lactic acid bacteria was determined after 72-hour incubation on MRS agar.

### Goblet cell count

The distal ileum was collected and washed with ice-cold PBS, followed by immediate immersion in 10% histological grade phosphate-buffered formalin (Mallinckrodt Chemical, Derbyshire, UK) for fixation. The fixed tissues were dehydrated in ethanol, embedded in paraffin wax, sectioned (5 μm thickness) and then stained with standard periodic acid-Schiff (PAS) stain. Five longitudinally well oriented villi and crypts were selected randomly from each mouse and the number of goblet cells were determined by counting PAS positive cells with a magenta color [13].

### Splenocyte isolation

The spleen of each mouse was removed and placed in 10 mL of RPMI-1640 medium (HyClone) supplemented with 1% antibiotics (50 μg/mL penicillin, 50 μg/mL streptomycin sulfate and 100 μg/mL neomycin sulfate, Invitrogen, Carlsbad, CA, USA). Each spleen was then cut into pieces with sterile operating scissors followed by pressing through a 70-μm cell strainer (BD Falcon, Bedford, MA, USA) under aseptic conditions. The released cells were rinsed with 10 mL RPMI-1640 medium containing 1% antibiotic and then centrifuged (800×g) at room temperature for 5 min. The cell pellet was collected and resuspended in 5 mL RBC lysis buffer (eBioscience, San Diego, CA, USA) for 2 minutes. After washing and centrifugation again, the cell pellet was next resuspended in 5 mL RPMI-1640 medium with 1% antibiotic and 10% heat-inactivated fetal bovine serum (FBS, Invitrogen). The number of cells in suspension was counted by Trypan blue (Invitrogen) assay, which was followed by incubation of the purified splenocytes at 37°C in a humidified atmosphere with 5% CO_2_.

### Toll-like receptor (TLR) agonist treatment

All TLR agonists were dissolved in endotoxin-free water or PBS supplied by manufacturer (Invivogen, San Diego, CA, USA). The isolated splenocytes (5×10^6^ cells) were treated separately with either a TLR2 (pam3csk4, 1 μg/mL), TLR3 (poly I: C, 1 μg/mL), TLR4 (lipopolysaccharide, 1 μg/mL), TLR5 (flagellin, 0.5 μg/mL) or TLR7/8 (R-848, 1 μg/mL) agonist for 24 hours. After treatment, each supernatant was collected by centrifugation (12,000×g, 5 minutes, 4°C) and stored in a −80°C freezer until required.

### Cytokine production

The cytokine levels in each supernatant after treatment with the agonists were measured on a DuoSet ELISA development system (R&D Systems, Minneapolis, MN) according to the manufacturer's instructions.

### Dextran sodium sulfate-induced colitis

The DSS-induced colitis model in mice was modified from the method of Wirtz *et al*. [14] and Chen *et al*. [9]. Four-week-old germ-free mice were separated into groups of six mice and each group was administrated with either control PBS or *L. kefiranofaciens* M1 2×10^8^ CFU intragastrically daily for 14 days at 2-day intervals. During the last 7 days of administration, 1% DSS (molecular weight: 36,000–50,000 Da;MP Biomedicals, Aurora, OH, USA) was added to the drinking water to induce colitis (Fig. 1B). The DSS solution was replaced with fresh DSS every 2. days. At the end of the treatment, the mice were anesthetized by isoflurane (Abbott Laboratories, Kent, UK) and then sacrificed by cervical dislocation. The length of each colon was measured and the feces in the colon were removed by flushing with ice-cold PBS. A 1-cm long fragment from the distal part of each colon was removed and immediately immersed in 10% histological grade phosphate-buffered formalin for fixation. The fixed colon fragments were dehydrated in ethanol, further embedded in paraffin wax, sectioned (5 μm thickness) and then stained with standard H&E stain. Histological evaluation of the intestines was carried out using the score system of Dieleman *et al*. [15] and evaluated by well-trained histologists. Briefly, the histological score was based on the level of tissue inflammation, which involved the extent of inflammation, the amount of tissue regeneration and the level of crypt damage in each intestinal tissue section. The intestinal bleeding score was calculated from the stool consistency and the level of intestinal bleeding. The occult blood in the feces was measured using a Hemoccult Sensa kit (Beckman Coulter, Brea, CA, USA).

### Statistical analysis

All values are given as mean ± standard deviation. All results were analyzed by Student's t-test or by one-way analysis of variance (ANOVA) followed by Duncan's multiple range test using Statistical Analysis System software (SAS Institute Inc., Cary, NC). A *p*-value <0.05 indicated that there was a significant difference.

## Results

### *Lactobacillus kefiranofaciens* M1 ameliorates cecal enlargement and stimulates intestinal epithelial cell growth in GF mice

**Figure 2:**
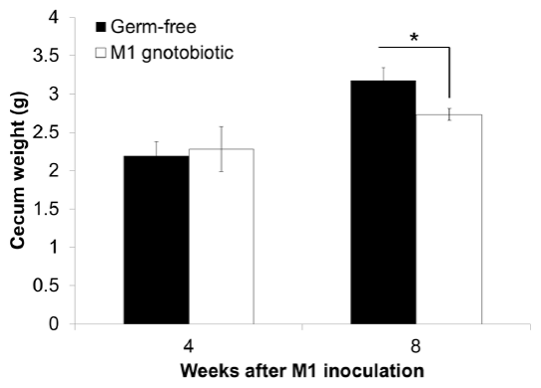
*Lactobacillus kefiranofaciens* M1 ameliorates cecal enlargement in germ-free mice. Four weeks old germ-free mice were single inoculated with live *L. kefiranofaciens* M1 (M1) intragastrically. The gnotobiotic mice associated with *L. kefiranofaciens* M1 and the parallel germ-free control mice were sacrificed after 4 and 8 weeks inoculation. The ceca were collected and weighted. (*p<0.05, Student's T-test).

We investigated the effects of the different administration periods of *L. kefiranofaciens* M1 on the intestines of GF mice. Cecal enlargement was observed in GF mice and increased with age (Fig. 2). Cecum weight decreased when the GF mice were singly inoculated with *L. kefiranofaciens* M1 when they were measured at the 8 week time point (SI-8w group); while, no significant difference was found between the gnotobiotic and GF control group at the 4 week time point (SI-4w group). These findings indicated that *L. kefiranofaciens* M1 was able to ameliorate the cecal enlargement of germ-free mice.

**Figure 3:**
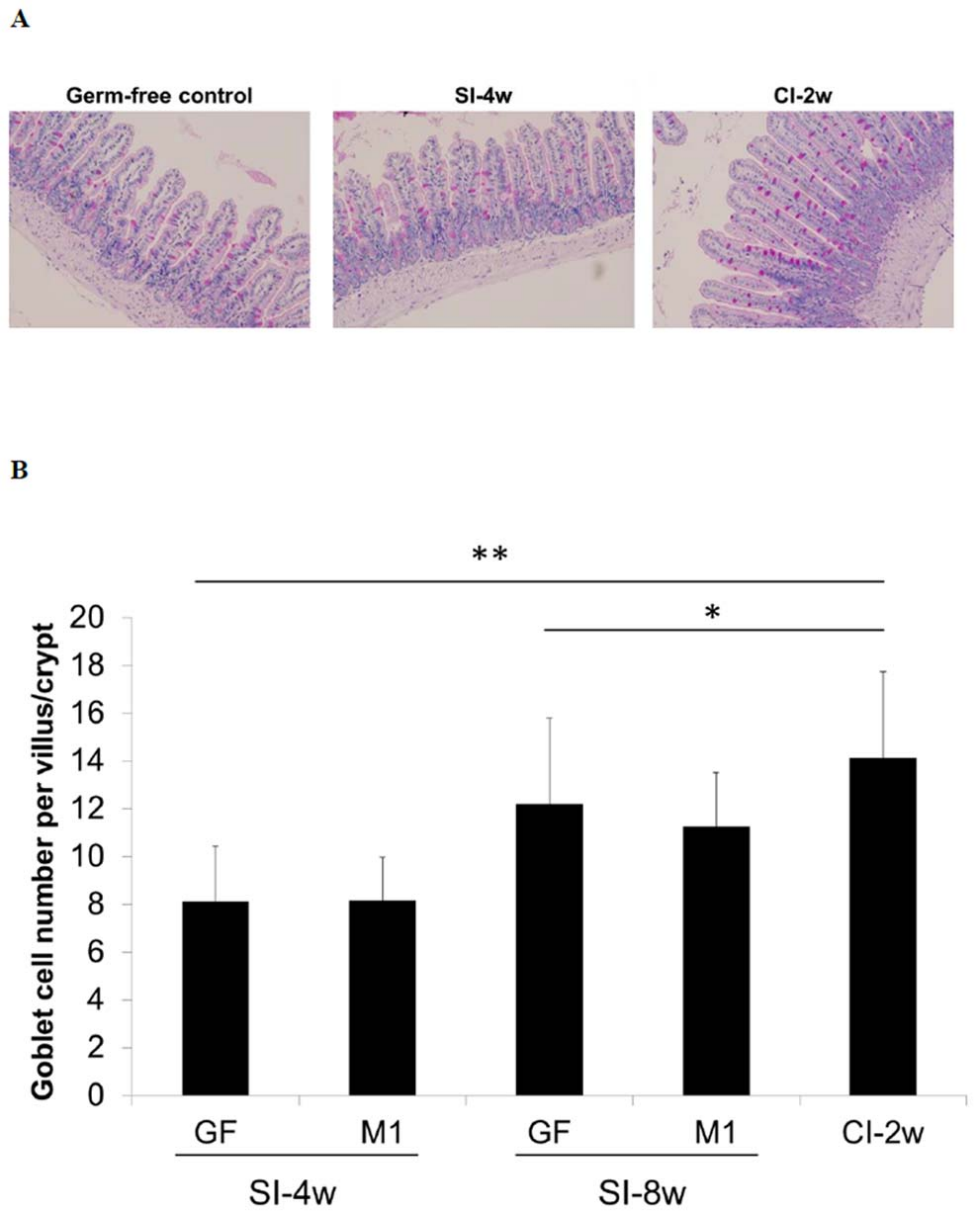
*Lactobacillus kefiranofaciens* M1 (M1) increases length of crypt-villus axis and goblet cell number in ileum of germ-free (GF) mice. Distal ileum sections were stained by periodic acid-Schiff (PAS) stain for determining goblet cell on villi. Representative histological photographs (A) and quantitative goblet cell numbers (B) were shown. (Germ-free control: 8 weeks old germ-free mice control; SI-4w: 4 weeks old germ-free mice after inoculation with *L. kefiranofaciens* M1 for 4 weeks; SI-8w: 4 weeks old germ-free mice after inoculation with *L. kefiranofaciens* M1 for 8 weeks; CI-2w: 4 weeks old germ-free mice after 2 weeks continuous inoculation with *L. kefiranofaciens* M1 in a 2-day interval) (n = 20–35, *p<0.05, **p<0.01, Student's T-test).

Next we evaluated the villus-crypt axis and goblet cells in the ileum of the GF mice. The results showed that continuous oral administration of *L. kefiranofaciens* M1 for 2 weeks (CI-2w group) was able to increase the ileac villus length and crypt depth of the GF mice as compared with the GF mice control group and the single inoculation group (SI-4w group) at the 4 week time point (Fig 3A). When the goblet cells in these mice were examined, it was observed that the number of goblet cells increased with age (Fig 3B). Furthermore, the continuous inoculation group had significantly higher numbers of goblet cells than other two groups. These findings demonstrated that *L. kefiranofaciens* M1 is able to stimulate intestinal epithelial cell growth and goblet cell differentiation.

### *Lactobacillus kefiranofaciens* M1 ameliorates DSS-induced colitis in GF mice

**Figure 4:**
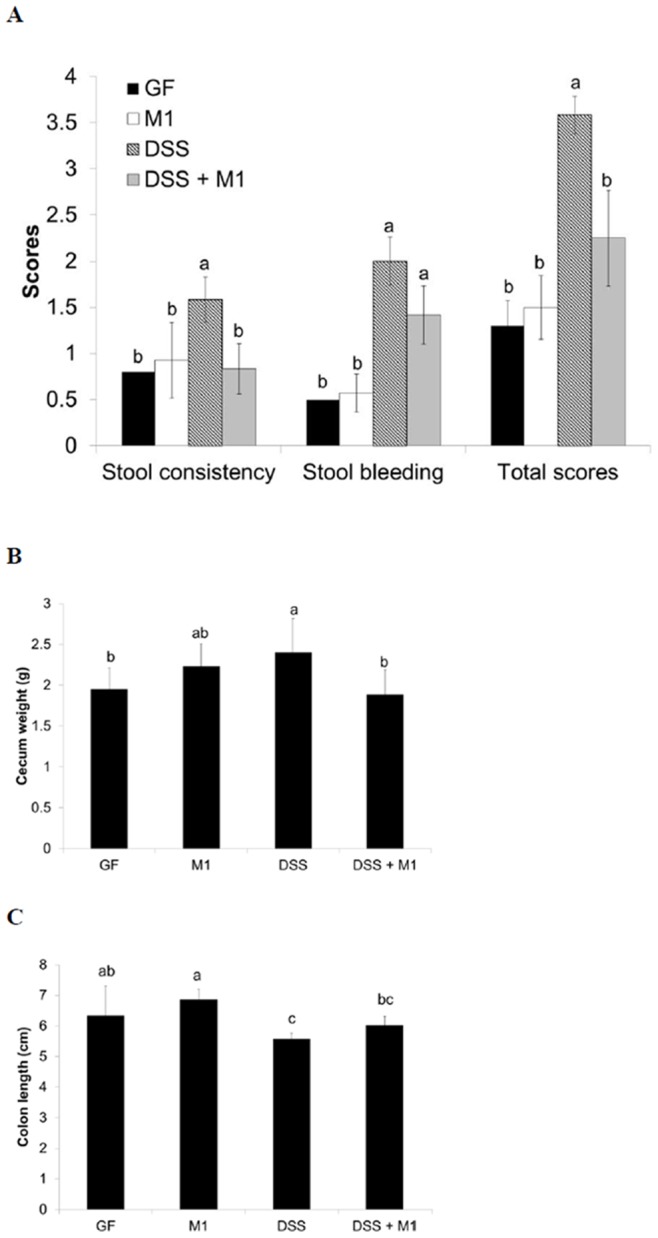
Effects of *Lactobacillus kefiranofaciens* M1 (M1) continuous inoculation on intestinal bleeding score (A), cecum weight (B) and colon length (C) in germ-free (GF) mice treated with dextran sodium sulfate (DSS). Means for data without a common letters differ significantly (*p*<0.05).

Next, the effects of administration with *kefiranofaciens* M1 on DSS-induced colitis in GF mice were examined. The results showed that the GF mice that had been continuously inoculated with *L. kefiranofaciens* M1 for two weeks had a lower intestinal bleeding score (Fig. 4A) and showed less cecal enlargement (Fig. 4B) when the DSS-induced colitis model mice were compared the GF control mice. Colon shortening was also found to have significantly improved in the continuous inoculation group (Fig. 4C).

**Figure 5:**
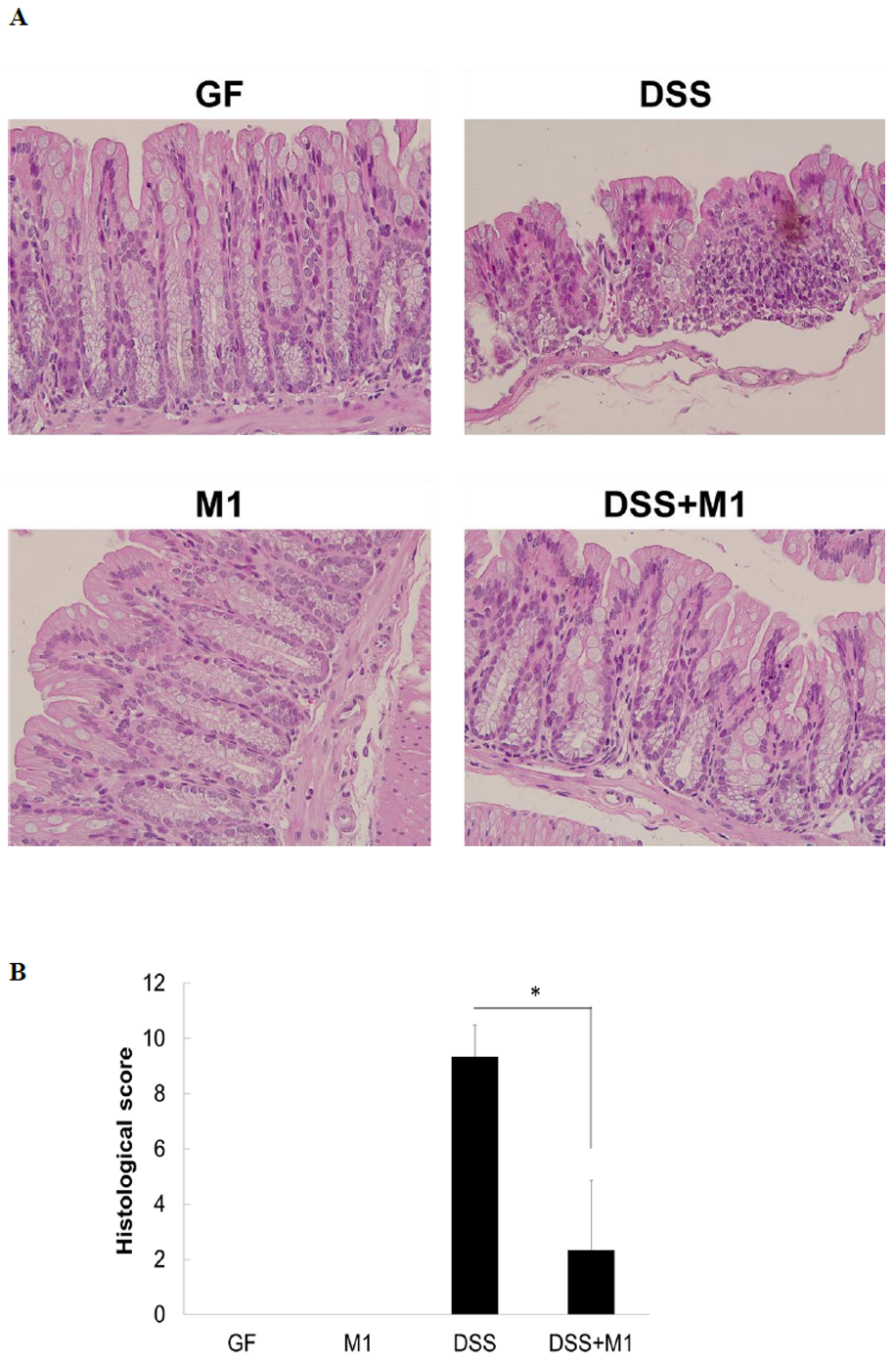
Representative histological photographs (A) and quantification of histological scores (B) of colon sections from non-treated or dextran sodium sulfate (DSS) treated germ-free (GF) mice control or *Lactobacillus kefiranofaciens* M1 gnotobiotic mice (M1) after continuous inoculation. Colon sections were evaluated and histological scores were quantitated after H&E staining. (*p<0.05, Student's T-test).

Histological analysis showed that the colitis control animals displayed signs of severe colitis, namely a high degree of inflammatory infiltrate in the colonic mucosa, a loss of goblet cells and a disturbed mucosal architecture. In contrast, inflammation was significantly reduced in the mice that had undergone continuous inoculation with *L. kefiranofaciens* M1 (Fig. 5A) and this was reflected in the fact that there was an improvement in the inflammatory score from 9 for the colitis control mice to 2 for the continuous inoculation mice (Fig. 5B).

### *Lactobacillus kefiranofaciens* M1 enhances TLR agonist-induced Th1 cytokine responses in splenocyte

**Figure 6:**
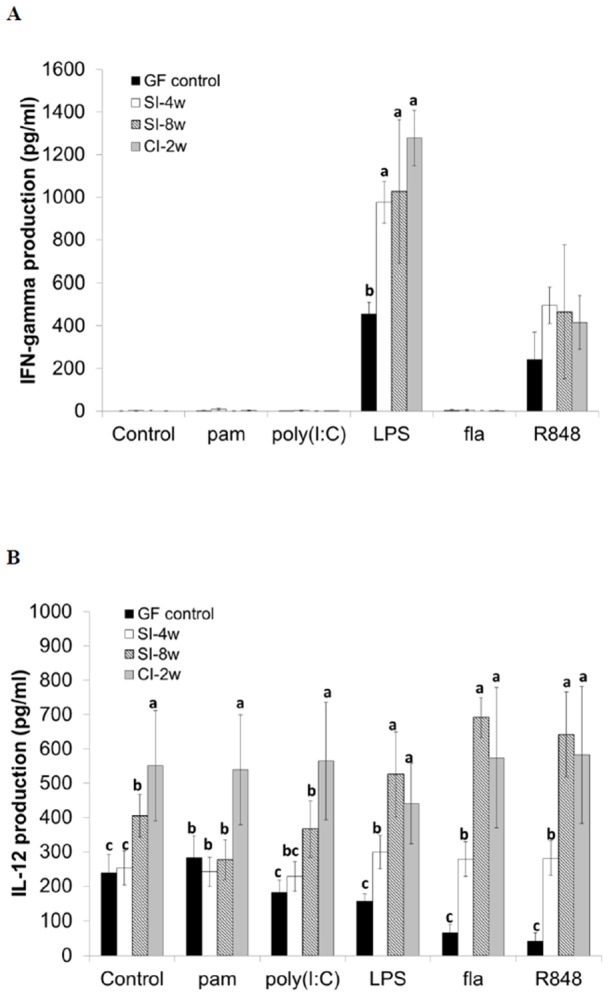
Effects of *Lactobacillus kefiranofaciens* M1 inoculation on splenic Th1 cytokines interferon (IFN)-γ (A) and interleukin (IL)-12 (B) responses stimulated by different Toll-like receptor (TLR) agonists in germ-free mice. The splenocytes of germ-free control mice (GF), gnotobiotic mice single inoculated with *L. kefiranofaciens* M1 (M1) after 4 and 8 weeks and continuous inoculation for 2 weeks were isolated. The isolated splenocytes were stimulated by TLR2 (pam: pam3csk4), TLR3 (poly(I: C)), TLR4 (LPS: lipopolysaccharide), TLR5 (fla: flagellin), TLR7/8 (R-848) agonists or left untreated. After 24 hours incubation, the supernatants were collected and the cytokine productions were measured. Means for data without a common letters differ significantly (*p*<0.05). (Germ-free control: 8 weeks old germ-free mice control; SI-4w: 4 weeks old germ-free mice after inoculation with *L. kefiranofaciens* M1 for 4 weeks; SI-8w: 4 weeks old germ-free mice after inoculation with *L. kefiranofaciens* M1 for 8 weeks; CI-2w: 4 weeks old germ-free mice after 2 weeks continuous inoculation with *L. kefiranofaciens* M1 in a 2-day interval).

The production of cytokines determines the type of immune response. Except for the TLR4 agonist LPS and the TLR7/8 agonist R848, no splenic IFN-γ production was observed with or without TLR agonist stimulation in all treatments. After mice had been inoculated with *L. kefiranofaciens* M1, exposure to LPS or R848 for 24 hours significantly induced splenic IFN-γ production as comparing with the GF mice control. On the other hand, IL-12 could be induced across all treatment groups with or without TLR agonist stimulation. IL-12 production was significantly higher in the GF mice that had been inoculated with *L. kefiranofaciens* M1. It is worth noting that the highest production of IFN-γ and IL-12 after TLR activation was detected in continuous group (CI-2w group) (Fig. 6), which indicates that continuous administration of this probiotic strain is necessary.

### *Lactobacillus kefiranofaciens* M1 is unable to colonize in GF mice

**Figure 7:**
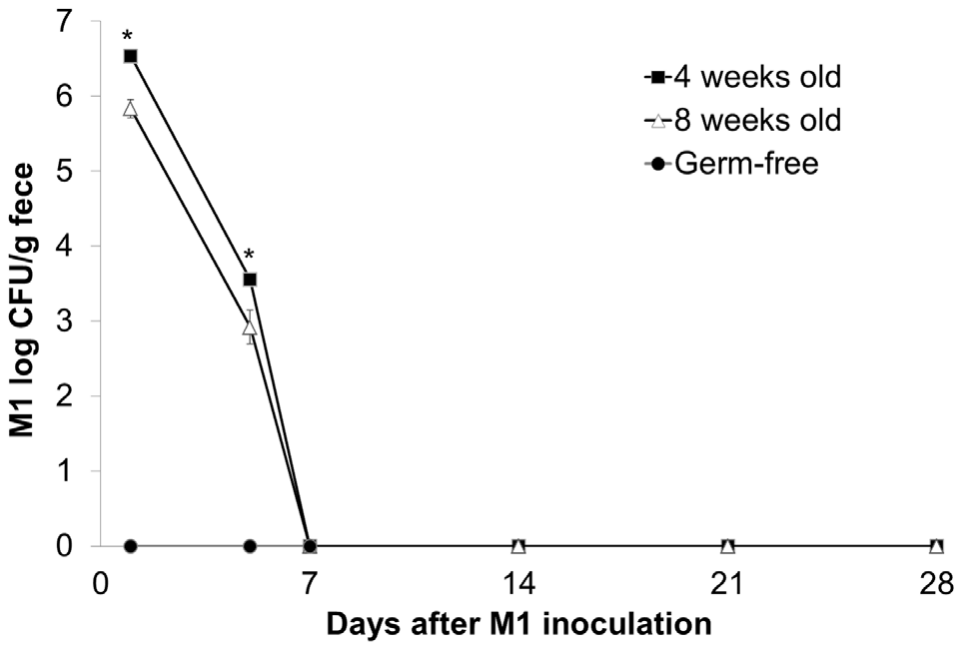
*Lactobacillus kefiranofaciens* M1 could not colonize in intestine of germ-free mice. Four and eight weeks old germ-free mice were single inoculated with live *L. kefiranofaciens* M1 (M1) intragastrically. The feces were collected after 1, 5, 7, 14, 21 and 28 days after inoculation and numbers of *L. kefiranofaciens* M1 in feces were counted. (CFU: colony-forming unit; *p<0.05, Student's T-test).

To determine whether *L. kefiranofaciens* M1 is able to colonize GF mice, microbiological analysis of the mouse feces from the various groups was carried out for 28 days. The findings indicated that *L. kefiranofaciens* M1 could be detected in the feces after a single oral innoculation at the 1 day time point, but the bacterial count had significantly drop by the 5 day time point. Furthermore, *L. kefiranofaciens* M1 could no longer be detected at the 7, 14, 21 and 28 day time points (Fig. 7). The bacterial count of *L. kefiranofaciens* M1 in feces of 4 weeks old mice was higher than that of 8 weeks old mice at both 1 and 5 days after single inoculation with *L. kefiranofaciens* M1 (Fig. 7). The gradually reduction of *L. kefiranofaciens* M1 numbers after inoculation indicated that *L. kefiranofaciens* M1 was unable to colonize the intestines of the GF mice. Additionally, *L. kefiranofaciens* M1 was found to not be able to bind to intestinal epithelial cells in an *in-vitro* adhesion test using Caco-2 cells (data not shown).

## Discussion

In this study, we used a germ-free mice model to elucidate whether the immunoregulation and anti-colitic ability of *L. kefiranofaciens* M1 directly work on the host or act via modulation of intestinal microbiota. Initially, the effects of oral administration of *L. kefiranofaciens* M1 on defects of intestinal function in the GF mouse, such as cecal enlargemtnt and reduction of crypt-villus length, were investigated. Cecal enlargement, which is caused by a lack of microbiota that remove accumulating mucus [16], is one of the most important feature of GF mice. Dysbiosis mice treated with antibiotics are also found to have similar symptoms [17]. Oral administration of *L. kefiranofaciens* M1 was able to ameliorate cecal enlargement in the GF mice at the 8 week time point, which indicates that this bacterium is able to relieve mucus accumulation, even though permanent colonization does not seem to occur.

Reduction in crypt depth and villus height in the small intestine is another important feature of GF mice. These phenomena are related to fewer proliferating stem cells in the crypts and slower enterocyte migration into the villus, which results in a lower enterocyte turnover rate [18]–[20]. Continuous administration of *L. kefiranofaciens* M1 was able to increase both ileac villus height and crypt depth in the GF mice. Other studies of probiotic strains such as *L. rhamnosus* GG [21] and *L. reuteri* DSM 17938 have also reported that they have a positive influence on crypt depth and villus height. It seem likely that the underlying mechanisms might involve stimulation of enterocyte migration and proliferation by the probiotic bacterial strain [22].

GF mice have a thinner mucus layer and fewer goblet cells than conventional mice [19], [23]. Goblet cells are important for the secretion of protective mucins and trefoil factors that help to maintain the mucosal barrier [12], [24], [25]. In the current study, we indicated that *L. kefiranofaciens* M1 may participate in intestinal homeostasis and defense against pathogens based on the finding that continuous inoculation of *L. kefiranofaciens* M1 was able to increase ileac goblet cell numbers in GF mice. Petersson *et* *al.* [26] reported that administration of a TLR2 agonist peptidoglycan was able to increase colonic mucus secretion by goblet cells in GF mice. Thus, we can hypothesize that TLR2, the cellular receptor of *L. kefiranofaciens* M1 [9], might also participate in improving goblet cell function in GF mice.

The effects of *L. kefiranofaciens* M1 on DSS induced colitis GF mice was also investigated. *L. kefiranofaciens* M1 treatment was found to ameliorate DSS-induced colitis in GF mice. In our early study, we have demonstrated that this strain is able to strengthen epithelial barrier function *in vitro* by increasing transepithelial electrical resistance (TEER) and significantly upregulated the level of the chemokine CCL-20 [9]. In addition, the increase in goblet cell numbers as a result of oral administration of *L. kefiranofaciens* M1 is likely to be very important for maintaining the mucosal barrier [26]. Ukena *et al*. [27] indicated that gnotobiotic mice mono-associated with probiotic *Escherichia coli* Nissle 1917 showed reduced symptoms of DSS-induced colitis via an increase in the tight-junction protein ZO-1 and enhanced mucosal integrity. Therefore, reinforcing/restoring epithelial barrier functionality might be a possible mechanism whereby *L. kefiranofaciens* M1 is able to ameliorate DSS-induced damage.

The production of cytokines via TLR agonist treatment was evaluated in order to determine the type of immune response that is active during the present study. The production of the inflammatory cytokines, IFN-γ and IL-12, was augmented after TLR agonist treatment in the continuous group (CI-2w group). The activated TLRs that were tested in this study was TLR2, TLR3, TLR4, TLR5 and TLR7/8, which mimic the various major ligand binding events in nature; these are peptidoglycan from Gram-positive bacteria, bacterial or viral double strand RNA, lipopolysaccharide from Gram-negative bacteria, bacterial flagellin and viral single strand RNA, respectively [28]. Sustained IL-12 signaling is essential for Th1 cell differentiation [29]. The present of IL-12 may also be responsible for the induction of IFN-γ expression [30]. Moreover, pathogens may be armed with several TLR agonists and may inspire different TLRs consecutively and in distinct cellular compartments. Amplifying production of inflammatory cytokines is linked to be anti-infection in nature, which explains that why the administration of *L. kefiranofaciens* M1 is able to inhibit enterohemorrhagic *Escherichia coli* infection *in vivo* (unpublished data). dos Santos *et al*. [31] also indicated that enhancement of IFN-γ production in splenocytes by feeding *L. delbrueckii* UFV-H2b20 is able to protect against *Listeria monocytogenes* infection in germ-free mice.

In addition to anti-infection activity, the production of the IFN-γ and IL-12 via TLR agonists in the continuous group is also important with respect to anti-allergic effects. Differential activation of TLRs in an allergic state can alter Th1/Th2 cytokine profiles [32], [33]. Importantly, GF mice are relatively sensitive to allergen-induced allergic airway inflammation due to their lack of microbiota colonization [34], [35]. An enhancement of the IFN-γ and IL-12 production via TLR agonists during the continuous administration of *L. kefiranofaciens* M1 is similar to the results of our previously report, which showed that administration of *L. kefiranofaciens* M1 is able to skew the Th1 response *in vitro* as well as in allergic and asthmatic conventional mice *in vivo* [7], [8], [10].

However, we found that *L. kefiranofaciens* M1 is unable to permanently colonize the intestine of GF mice. In addition, *L. kefiranofaciens* M1 does not seem to be able to bind to intestinal epithelial cells based on the results of an *in-vitro* adhesion test using Caco-2 cells that was carried out. The lack of colonization ability might be due to the exopolysaccharide producing properties of *L. kefiranofaciens* [36]. Bacterial exopolysaccharides are known to reduce the probiotic adhesion ability of bacteria *in vitro*; it has been reported that a high exopolysaccharide producing strain of lactobacillus was found to have almost lost its adhesion ability compared to the parallel strains with same genetic background that had normal exopolysaccharide production [37], [38]. The lack of colonization of the intestine explained why continuous feeding *L. kefiranofaciens* M1 is able to produce better effects in terms of the generation and maintenance of immunoregulation and, furthermore, was able to stimulate the highest Th1 cytokine responses. It worth noting that the probiotic effects of non-colonizing *L. kefiranofaciens* M1 on GF mice persisted even 4 or 8 weeks after a single inoculation. Hapfelmeier *et* *al*. [39] reported a similar finding for a non-colonizing *E. coli* strain and showed that the initial bacterial loading was more important than colonization.

## Conclusion

In the present study, we proved that the probiotic bacterium *L. kefiranofaciens* M1 is able to act directly on the mouse host without intestinal microbiota and enhance immunoregulation and intestinal functionality. Continuous consumption of *L. kefiranofaciens* M1 to increase the initial bacterial loading in the intestine might be necessary to maintain its probiotic functions. To the best of our knowledge, this is the first paper using a DSS-GF mice model to evaluate the anti-colitis effects of a probiotic *Lactobacillus* sp.
